# An interpretable machine learning framework for diagnosis and prognosis of COVID-19

**DOI:** 10.1371/journal.pone.0291961

**Published:** 2023-09-21

**Authors:** Yongxian Fan, Meng Liu, Guicong Sun

**Affiliations:** School of Computer Science and Information Security, Guilin University of Electronic Technology, Guilin, 541004, China; Abdul Wali Khan University Mardan, PAKISTAN

## Abstract

Coronaviruses have affected the lives of people around the world. Increasingly, studies have indicated that the virus is mutating and becoming more contagious. Hence, the pressing priority is to swiftly and accurately predict patient outcomes. In addition, physicians and patients increasingly need interpretability when building machine models in healthcare. We propose an interpretable machine framework(KISM) that can diagnose and prognose patients based on blood test datasets. First, we use *k*-nearest neighbors, isolated forests, and SMOTE to pre-process the original blood test datasets. Seven machine learning tools Support Vector Machine, Extra Tree, Random Forest, Gradient Boosting Decision Tree, eXtreme Gradient Boosting, Logistic Regression, and ensemble learning were then used to diagnose and predict COVID-19. In addition, we used SHAP and scikit-learn post-hoc interpretability to report feature importance, allowing healthcare professionals and artificial intelligence models to interact to suggest biomarkers that some doctors may have missed. The 10-fold cross-validation of two public datasets shows that the performance of KISM is better than that of the current state-of-the-art methods. In the diagnostic COVID-19 task, an AUC value of 0.9869 and an accuracy of 0.9787 were obtained, and ultimately Leukocytes, platelets, and Proteina C reativa mg/dL were found to be the most indicative biomarkers for the diagnosis of COVID-19. An AUC value of 0.9949 and an accuracy of 0.9677 were obtained in the prognostic COVID-19 task and Age, LYMPH, and WBC were found to be the most indicative biomarkers for identifying the severity of the patient.

## Introduction

Since December 2019, coronaviruses have spread worldwide, affecting many people’s lives. Globally, as of 5:48 pm, CET on November 2, 2022, COVID-19 cases have been reported to WHO, including 6,572,800 deaths. As of October 31, 2022, 6,283,553 have been confirmed cases [1]. Coronaviruses pose a massive threat to the health and lives of people worldwide. However, the large number of asymptomatic individuals shown by COVID-19 symptomatology makes differentiating between COVID-19 positive and negative individuals a challenge [2]. At the same time, the severity of SARS-CoV-2 pneumonia puts considerable pressure on intensive care resources (ICU) in hospitals without adequate resources [3]. Therefore, it is believed that a correct diagnosis of COVID-19 and a correct prognosis based on COVID-19 severity will be crucial for pandemic containment [4].

The clinical diagnosis of COVID-19 is based on clinical presentation, reverse transcriptase polymerase chain reaction (RT-PCR) [5], molecular diagnosis of the viral genome by chest X-ray or CT scan [6,7], and serologic blood tests. Most countries have opted for reverse transcriptase polymerase chain reaction testing of respiratory samples. But the high demand for RRT-PCR testing has exposed the limitations of this diagnostic on a large scale, such as long turnaround times [8,9], the need for trained personnel, expensive equipment, and demand for reagents outstripping supply [10]. The rapid antigen test is a screening test that identifies COVID-positive patients within 15 minutes but is less sensitive than the PCR test [11]. Therefore, an urgent search for other inexpensive and more accessible tests or complementary methods is essential.

On the other hand, although more than 80% of patients with COVID-19 are mild to moderate cases, approximately 14% of patients are severely ill, and 5% are critically ill [1]. Critically ill cases often develop acute respiratory distress syndrome (ARDS) or multiple organ dysfunction syndromes (MODS) within just two weeks of infection [12], consuming most of the healthcare resources and leading to high morbidity and mortality rates (up to 49%) [13]. Early prediction of COVID-19 severity can help to rapidly classify patients (isolation, hospitalization, ICU assignment, etc.) and optimize the use of medical resources and timely medical intervention [14,15]. There is an intense interest in using machine learning tools to combat the COVID-19 pandemic, including disease diagnosis, prediction, prevention, treatment, and prognosis. Davide Brinati et al. demonstrated the feasibility of applying routine, low-cost blood tests to machine learning studies and evaluating predictive models in large-scale screening of potentially COVID-19-infected individuals [16]. Several clinical studies have shown that COVID-19 patients exhibit considerable variation in blood parameters and that the identification of these parameters can play a vital role in the initial screening for COVID-19 [17–20]. It isn’t easy even for experienced physicians to fully extract all the information in routine blood tests. However, machine learning algorithms can effectively learn and distinguish between the various associations observed in the parameters of regular blood tests. As a result, some initial efforts have begun to develop artificial intelligence methods for the diagnosis of COVID-19 and mortality from regular blood samples [19,21–23]. In addition, there are also some research methods utilizing novel approaches to predict disease diagnosis [24–26]. However, research efforts are still in their infancy.

Explainable machine learning refers to machine learning models that can explain why specific predicted results are made. In healthcare, there is a need for more than just delivering traditional machine learning metrics. Furthermore, clinical providers and other decision-makers prioritize the interpretability of the model’s underlying predictive patterns [27,28]. Physicians and others involved need to have confidence in the model’s predictions for such important decisions [29]. There is a close connection between interpretability and feature importance in machine learning. Feature importance helps us understand which features in the model have a greater influence on the prediction results. By analyzing feature importance, we can determine which features are key factors in the model’s decision-making process, thereby aiding our understanding of the model’s prediction process. Feature importance provides an explanation for the model’s prediction results. When a feature has higher importance, we can infer that it contributes more to the prediction results. This inference helps us understand why the model makes specific predictions and enhances trust and reliability in the model’s predictions.

In this study, we propose an interpretable machine learning framework KISM for the diagnosis and prognosis of COVID-19. First, we perform data pre-processing. Among them, we utilize the K-Nearest Neighbors Imputer(KNNImputer) algorithm to handle null values, employ the isolation Forest (iForest) for outlier detection, and then apply the Synthetic Minority Over-Sampling Technique (SMOTE) [30] for data distribution balancing. Second, machine learning algorithms- Support Vector machine(SVM), Extra Tree (ET), Random Forest(RF), Gradient Boosting Decision Tree(GBDT), eXtreme Gradient Boosting (XGB), Logistic Regression(LR), and ensemble model(REGX) are used. Finally, feature importance is reported by Feature_Importances [31] and SHapley Additive exPlanations (SHAP) [32] to meet the need for model interpretability in the healthcare setting. We conduct comparative experiments on two benchmark datasets.

## Materials and methods

### Method overview

**Fig 1:**
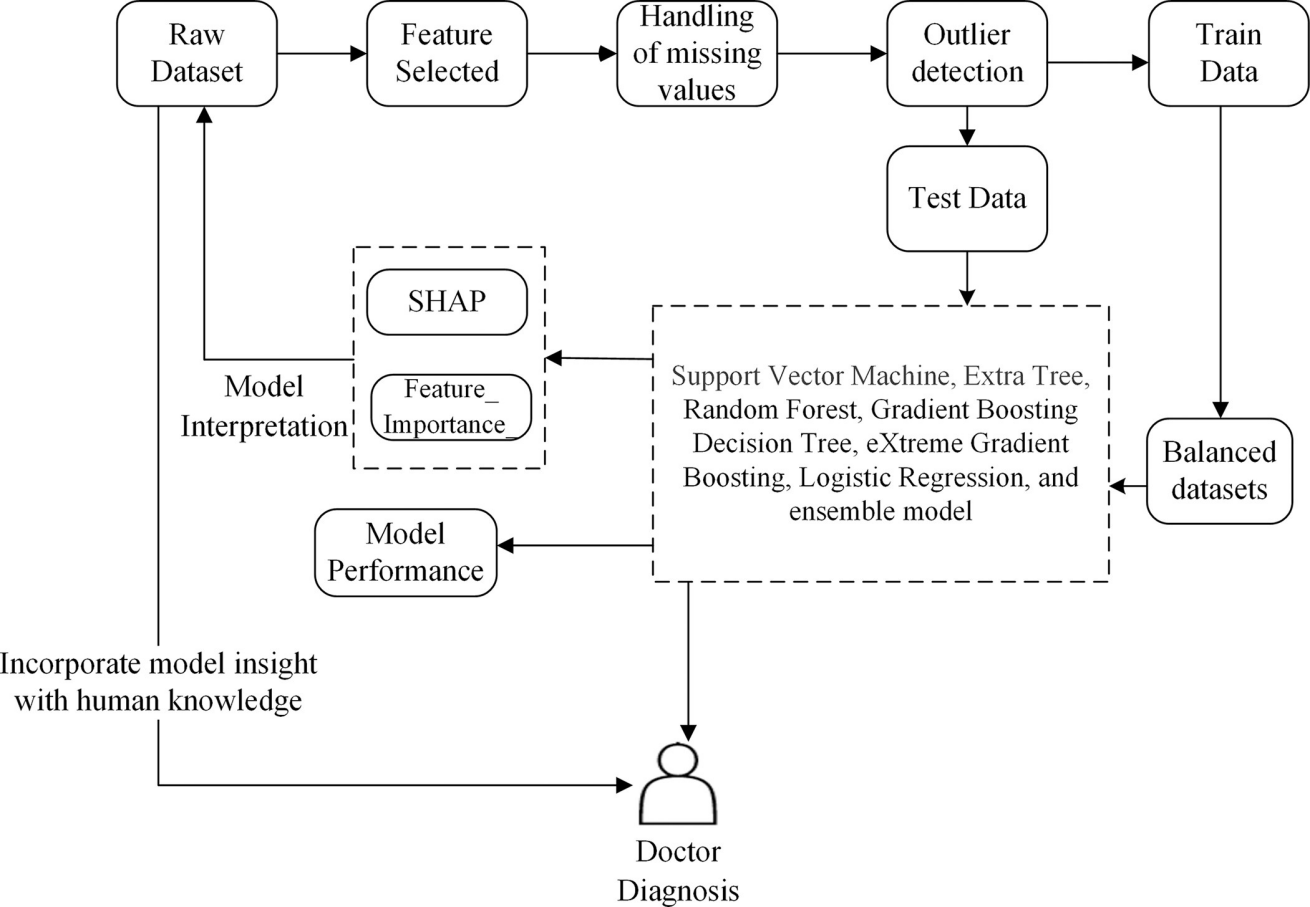
Framework of KISM for COVID-19 diagnosis and prognosis.

In this section, we introduce KISM, a machine learning framework for the diagnosis and prognosis of COVID-19 that is designed to be interpretable. The framework utilizes various machine learning algorithms including Support Vector Machine (SVM), Extra Tree (ET), Random Forest (RF), Gradient Boosting Decision Tree (GBDT), eXtreme Gradient Boosting (XGB), Logistic Regression (LR), and ensemble learning. The framework is shown in Fig 1.

SVM is a binary classification algorithm that aims to find a hyperplane to separate the samples. The goal is to maximize the distance between the line and the nearest sample points of the two categories.

ET is an integrated learning technique that combines the results of multiple decision trees collected in a forest to output classification results. Each decision tree in the extreme random tree is constructed from the original training samples.

RF is a model composed of multiple decision trees. Instead of building a large decision tree with the entire training dataset, RF constructs several small decision trees with different subsets of data and characteristic attributes. These trees are then merged to create a more powerful model.

GBDT is an iterative decision tree algorithm that constructs a set of weak learners (trees). The results of multiple decision trees are added up to produce the final prediction output.

XGB is an integrated machine learning algorithm based on decision trees. It uses a gradient-boosting framework and is suitable for classification and regression problems.

LR is a classification and prediction algorithm that predicts the probability of future results based on the performance of historical data.

**Fig 2:**
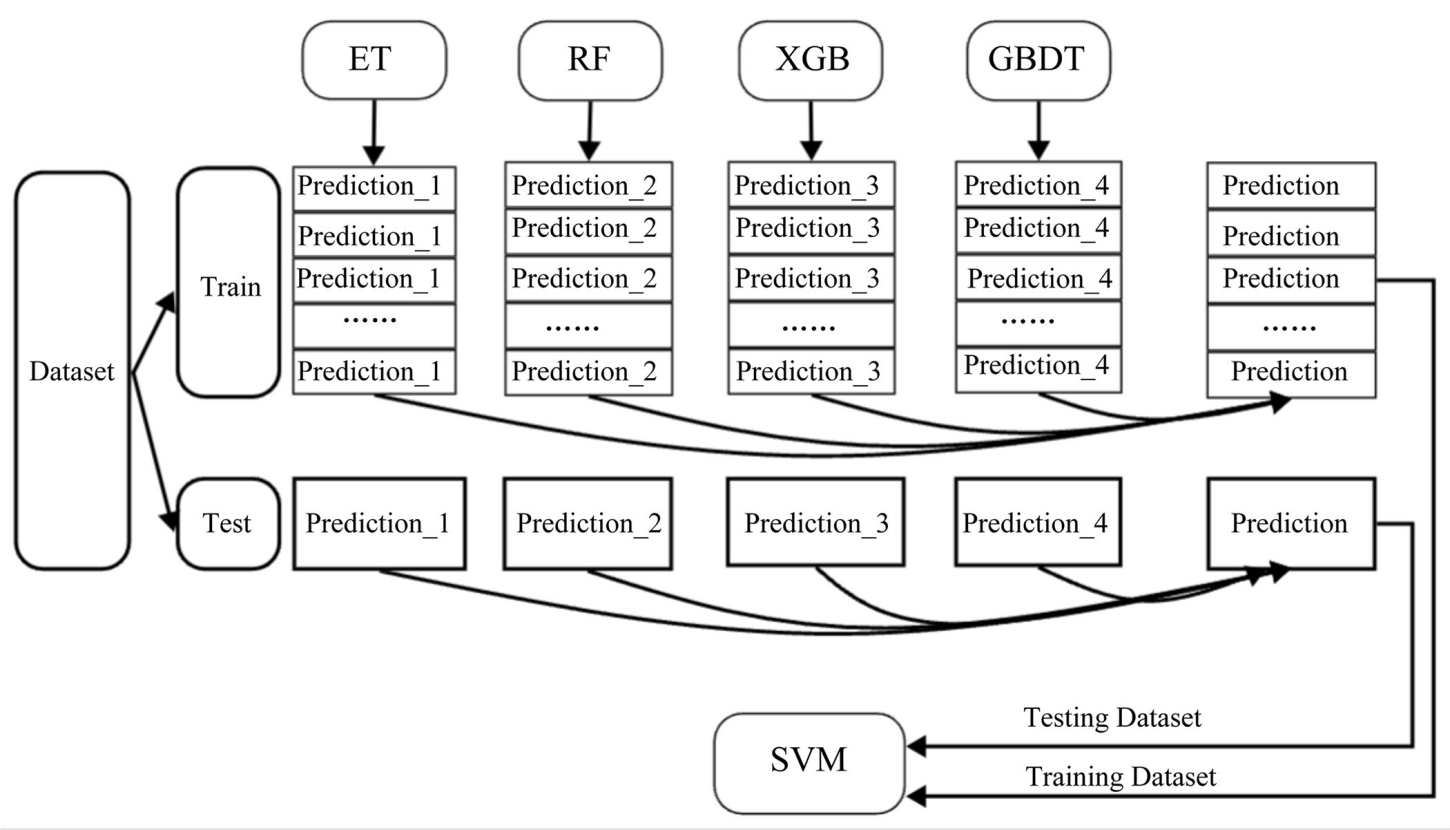
A flowchart of the internal running of the REGX model.

Ensemble learning(REGX) is a machine learning method that combines the results of multiple learners to achieve better learning results than a single learner. In this framework, we use a two-stage classifier, where the output of the first-stage classifier (RF, ET, GBDT, XGB) is fed to the second-stage classifier (SVM) to improve the overall classifier performance. In addition, The name for this ensemble learning comes from the first-stage classifier model. The specific training process is shown in Fig 2.

Then, to explain the model, this paper uses Feature_ Importance_ and SHAP to evaluate the contribution of each feature to the prediction as a way to show some vital feature markers that physicians may overlook.

For the parameter settings, all the parameters in this paper use the grid search algorithm. Improper selection of hyperparameters can lead to underfitting and overfitting of the model. The grid search can be well used to optimize the model performance by traversing the given parameter combinations, which is a training and comparison process. The selected hyper-parameter values are as follows in Table 1.

**Table 1:** Hyper-parameter values of the KISM.

Dataset	Model	parameter values
Dataset1	SVM	probability = True;
	ET	n_estimators = 300, max_depth = 17;
	RF	n_estimators = 300, max_depth = 17;
	GBDT	learning_rate = 0.1, n_estimators = 290, min_samples_split = 200, min_samples_leaf = 20, max_depth = 8, max_features = ’sqrt’, subsample = 0.8, random_state = 10;
	XGB	learning_rate = 0.4, n_estimators = 50, max_depth = 17, eval_metric = ’error’;
	LR	solver = ’liblinear’, max_iter = 200, multi_class = ’ovr’, class_weight = ’balanced’;
	REGX	SVM: probability = True; ET: n_estimators = 300, max_depth = 17; RF: n_estimators = 300, max_depth = 17; GBDT: learning_rate = 0.1, n_estimators = 290, min_samples_split = 100, min_samples_leaf = 30, max_depth = 11, subsample = 0.85, random_state = 10; XGB: learning_rate = 0.4, n_estimators = 200, max_depth = 17 eval_metric = ’error’;
Dataset2	SVM	probability = True;
	ET	n_estimators = 300, max_depth = 15;
	RF	n_estimators = 100, max_depth = 19;
	GBDT	----
	XGB	n_estimators = 80, learning_rate = 0.45,max_depth = 7, eval_metric = ’error’;
	LR	solver = ’liblinear’, max_iter = 90, multi_class = ’ovr’, class_weight = ’balanced’;
	REGX	SVM: probability = True; ET: n_estimators = 300, max_depth = 15; RF: n_estimators = 100, max_depth = 19; GBDT: ---- XGB: n_estimators = 80,learning_rate = 0.45,max_depth = 7, eval_metric = ’error’;

To prevent model overfitting, ten-fold cross-validation was applied to all machine learning models in this study. For ensemble models, the first-level models were built using ten-fold cross-validation and data shuffling. The experimental results were evaluated based on 100 repetitions. In each repetition, a random sample of the dataset was taken, with 80% used for training and 20% for testing model performance. The average value and 95% confidence interval of these 100 repetitions were calculated using the randomly selected datasets. The sample size for each repetition was set to 80% of the entire dataset. You can find more detailed model information on our GitHub homepage. The specific URL is https://github.com/liumeng2026/bookish-guacamole/.

### Benchmark datasets

In this study, we evaluate the advantages of KISM on two authoritative datasets. The first dataset [33] was obtained from laboratory results of patients attending the Israel Albert Einstein Hospital in São Paulo, Brazil. It contains data from routine blood tests for both COVID-19 and non-COVID-19 cases. Following the work of Talha Burak Alakus et al, we selected 19 laboratory characteristics that are essential for the diagnosis of COVID-19 [34]. The final dataset contained ages and 18 laboratory test results for 600 patients. Of these patients, 520 had no findings, and 80 were COVID-19 patients. It contains 19 input features and one output feature.

The second dataset [35] was obtained from 196 COVID-19 patients diagnosed at Wuhan Red Cross Hospital between February 1, 2020, and March 15, 2020. The cases were classified as Mild-Moderate and Severe-Critically Severe [36]. The dataset contained 67 mild to moderate and 129 severe patients with eight input features and one output feature (Severity). For convenience, we denote the two datasets as Dataset1, and Dataset2, respectively. Detailed statistics are shown in Table 2.

**Table 2:** Details of the input and output characteristics of the COVID-19 datasets.

Datasets	Features	Number of features
Dataset1[33]	Patient age quantile, Hematocrit, Hemoglobin, Platelets, Red Blood Cells, Lymphocytes, Leukocytes, Basophils, Eosinophils, Monocytes, Serum Glucose, Neutrophils, Urea, Proteina C reativa mg/dL, Creatinine, Potassium, Sodium, Alanine Transaminase, Aspartate transaminase	19
Dataset2 [35]	Gender, Age, Leukocytes (WBC), lymphocyte count (LYNC), lymphocyte ratio (LYMPH), neutrophil count (NEUT), neutrophil ratio (NEU), neutrophil to lymphocyte ratio (NLR)	8

### Data preparation

Data pre-processing is essential in building machine learning models, and choosing the appropriate processing method helps reduce data redundancy and avoid noisy data, thus improving model performance. Preparing the data is divided into three steps.

#### (1) Handling of missing values

KNNImputer is used to estimate missing values using k-nearest neighbors to fill in missing values such as None. The missing values for each sample are calculated using the average of the n_neighbors nearest neighbors found in the training set.

#### (2) Outlier detection

We use iForest to remove outliers from the original data. iForest is suitable for detecting outliers in continuous data, where outliers are points that are sparsely distributed and far away from dense areas. In statistical terms, a sparse region in the data space means the probability of the data occurring in this region is low. Therefore, the data falling in these regions can be considered anomalous. Forest consists of isolated trees; each isolation tree is a binary tree structure. iForest is implemented in the following steps:

- Randomly select several sample points as subsamples from the training data and put them into the root node of the tree.
- A feature is randomly selected as a new node, and a cut point *P* is randomly generated in the current feature data: the cut point is generated between the maximum and minimum values of the specified dimension in the current node data.
- A hyperplane is generated with this cut point. Then the current node data space is divided into two subspaces: data less than *P* in the specified dimension is placed in the left child of the current node, and data greater than or equal to *P* is placed in the right child of the current node.
- Recursively execute the above two steps in the child nodes, and keep constructing new child nodes until there is only one data in the child nodes (no more cuts can be made), or the child nodes have reached the limited height of the tree.

After obtaining *T* isolation trees, the training of iForest is completed, and the generated iForest can be used to evaluate the test data. For every training data point *x*, it needs to traverse each isolation tree and determine the level at which *x* eventually resides in each tree. Thus, we can calculate the average path length of *x* in each tree, which allows us to derive the anomaly score for each instance based on the average path lengths.

In this paper, we adjust two parameters of iForest: n_estimators and contamination. After removing the outliers, the dataset is divided into an 80% training set and a 20% testing set.

#### (3) Balanced datasets

The Dataset1 contains 87% SARS-CoV-2 negative patients and about 13% SARS-CoV-2 positive patients. The Dataset2 contains 34% mild to moderate patients and 66% severe patients. The up-sampling and down-sampling strategies are primary methods to solve the class imbalance problem. Up-sampling means increasing the number of minority class samples, and down-sampling means decreasing the majority class samples. We use SMOTE to achieve class balancing, which does not directly resample the minority classes but designs algorithms to artificially synthesize some new minority samples. The synthesis strategy of SMOTE is as follows:

- For each sample *a* in the minority class, calculate its distance to all samples in the minority class sample set in terms of Euclidean distance to get its k-nearest neighbors.
- Set a sampling ratio according to the sample imbalance ratio to determine the sampling multiplicity *N*. For each minority class sample *a*, select several samples randomly from their k-nearest neighbors, assuming that the selected nearest neighbor is *b*.
- For each randomly selected nearest neighbor *b*, respectively, construct a new sample *c* with the original sample *a* according to the following formula:

c=a+rand(0,1)*|a−b| (1)

where rand(0,1) represents a randomly selected number between 0 and 1.

### Interpretability

Interpretability is not limited to machine learning models but also includes other aspects, such as intrinsic or ex-post classification, pre-model, intra-model, or post-model, and classification based on model results [37]. Explainable models provide essential insights into why predictions are made. However, they impose constraints on the model, representation(features), and user expertise. In contrast, model-independent explanatory systems provide a general framework for interpretability, allowing for flexible choices of model, representation, and user expertise [38]. Model-independent interpretability is critical in making machine learning more trustworthy and, ultimately, more beneficial [39].

An important step in classification models is the selection of appropriate features. The importance of a feature measures the magnitude and direction of the feature’s influence on the prediction, whether positive or negative. There are several methods used to determine feature importance. In this study, we use Feature_Importances_ and SHAP to evaluate the importance of features in predicting COVID-19.

**Fig 3:**
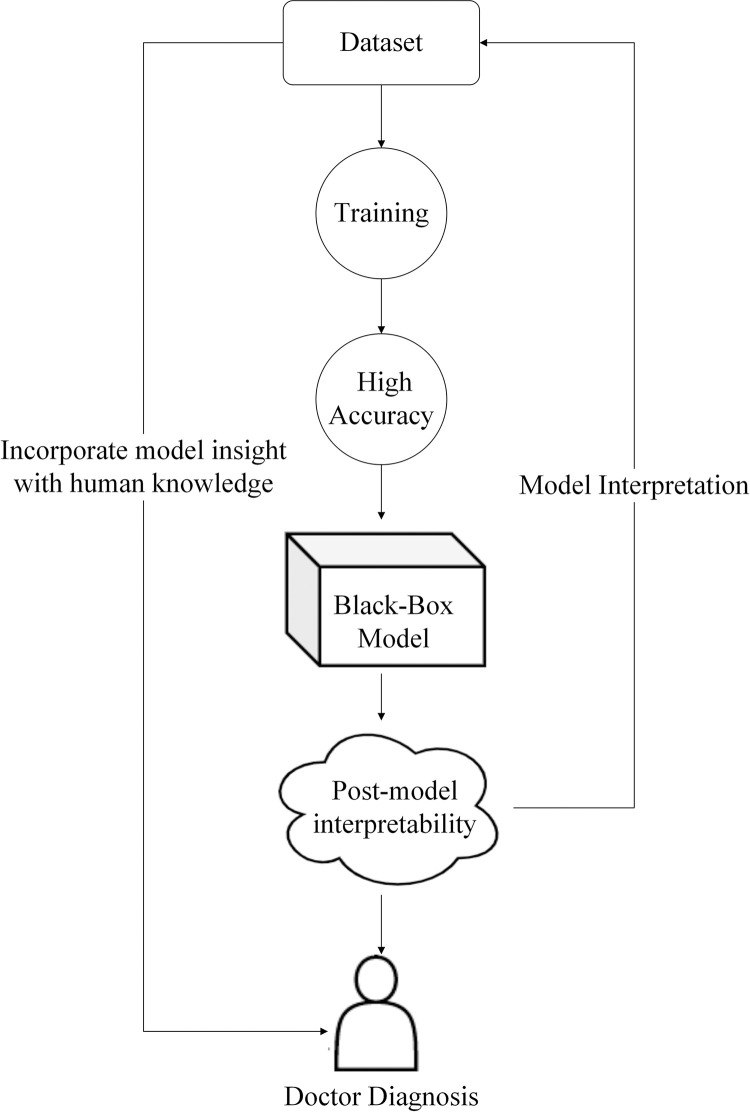
The interpretable model workflow used by KISM.

The Feature_Importances_ attribute in scikit-learn returns the importance of the features. As explained in the official scikit-learn documentation as follows: the importance of a feature is computed as the (normalized) total reduction of the criterion brought by that feature. The importance of features is calculated by looking at the splits of each tree. The basic idea is that the more times a feature is selected as a split, the more influential the feature is. Shapley’s value originates from game theory, while the meaning of SHAP is to observe the effect of each feature in predicting a specific sample on the prediction result. The basic idea is to calculate the marginal contribution of an element when it is added to the model and then take the average value of the different marginal contributions of the feature in the case of all the feature sequences, i.e. the SHAP baseline value of a specific feature. The SHAP value measures the marginal contribution of a feature and is one of the best methods for model interpretation. The interpretability workflow used by KISM is shown in Fig 3. First, the dataset is trained by a black box model to obtain high-precision conclusions. Then, the critical biomarkers inspected by the black-box model are revealed through post hoc interpretability. Finally, the professional medical stakeholders can combine the interpretable model insight results with their medical expertise to reveal the most critical indicators in early diagnosis on time. In conclusion, through model interpretation, black box models can reveal important biomarkers that physicians may have overlooked due to the surge of infected patients in the COVID-19 pandemic.

### Evaluation metrics

Appropriate model evaluation metrics are crucial to building a reliable model. In this work, we use objective evaluation metrics in machine learning: AUC, Accuracy, Precision, and Specificity. These performance metrics are calculated according to the confusion matrix. The confusion matrix contains information from four components.

- True Positive (TP): The number of positive samples that are correctly classified,
- False Positive (FP): The number of negative samples incorrectly labeled as positive samples.
- True Negative (TN): The number of negative samples that are correctly classified.
- False Negative (FN): The number of positive samples incorrectly labeled as negative samples.

As a result, multiple assessment metrics were defined as follows:

$$Accuracy=\frac{TP+TN}{TP+TN+FP+FN}$$ (2)

$$Specificity=\frac{TN}{TN+FP}$$ (3)

$$Sensitivity=\frac{TP}{TP+FN}$$ (4)

$$Precision=\frac{TP}{TP+FP}$$ (5)

Moreover, The Receiver Operating Characteristic (ROC) curve is a composite indicator of the sensitivity and specificity of continuous variables, and each point on the ROC curve reflects the perceptibility of the same signal stimulus. The value of AUC is the area under the ROC curve. The AUC visually reflects the classification ability expressed by the ROC curve. The closer the AUC value is to 1, the better the model performance.

## Results and discussion

### Comparison experiments

The performance of the developed models, in terms of Accuracy, AUC, Precision, Specificity, F1-score, Recall and Sensitivity is reported in Table 3. As shown in Table 3, although some of the models on Dataset1 and Dataset2 were higher than ensemble learning on a partial index, overall the ensemble learning, achieved optimal results.

**Table 3:** Comparison of different machine learning model methods.

Dataset	Model	Accuracy	AUC	Precision	Recall	F1-score	Sensitivity	Specificity
Dataset1	SVM	0.8617	0.9349	0.4476	0.8067	0.5688	0.8067	0.8688
	ET	0.9404	0.9689	0.8143	0.7762	0.7871	0.7762	0.9677
	RF	0.9340	0.9763	0.8172	0.8181	0.8101	0.8181	0.9588
	GBDT	0.9340	0.9711	0.7321	0.7933	0.7476	0.7933	0.9537
	XGB	0.9170	0.9398	0.6548	0.7262	0.6817	0.7262	0.9460
	LR	0.8617	0.9031	0.4736	0.8433	0.5888	0.8433	0.8631
	REGX	**0.9787**	**0.9869**	**0.8182**	**1.0000**	**0.9000**	**1.0000**	**0.9765**
Dataset2	SVM	0.7450	0.8352	0.9274	0.6718	0.7706	0.6718	0.9000
	ET	0.8688	0.9409	0.9363	0.8818	0.9059	0.8818	0.8350
	RF	0.8950	0.9495	0.9484	0.8867	0.9125	0.8867	0.9100
	GBDT	0.9283	0.9694	0.9600	0.9244	0.9402	0.9244	0.9333
	XGB	0.9083	0.9539	0.9344	0.9445	0.9360	0.9445	0.8150
	LR	0.8425	0.9157	0.8981	0.8292	0.9579	0.8292	0.8571
	REGX	**0.9677**	**0.9949**	**1.0000**	**0.9545**	**0.9767**	**0.9545**	**1.0000**

**Fig 4:**
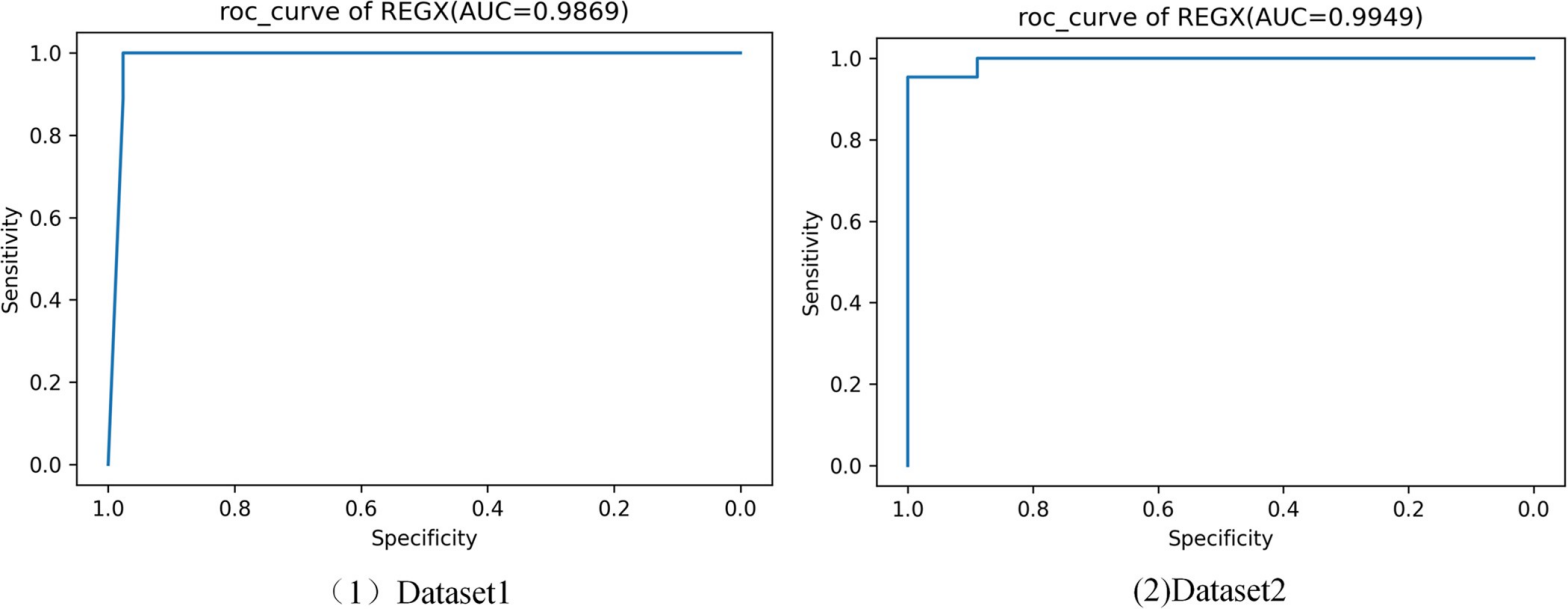
ROC curves of the REGX.

In addition, we performed ablation experiments to compare the performance of machine learning methods with different combinations of integrated learning, and the results are shown in Table 4. Ensuring that all other conditions remain the same and we only change the combination of different tree models. We can see that combining four machine learning algorithms to obtain the most superior performance on both datasets, where the Accuracy of 97.87% and the AUC of 98.69% were obtained on Dataset1, and the Accuracy of 96.77% and the AUC of 99.49%were obtained on Dataset2. The results of the ablation experiments show that each tree model is valid and that REGX obtains optimal performance by combining the four tree models. Finally, the ROC curve of the REGX model is shown in Fig 4.

**Table 4:** Ensemble model end different machine learning combination model comparison.

Dataset	Model	Accuracy	AUC	Precision	Recall	F1-score	Sensitivity	Specificity
Dataset1	RF, GBDT, XGB	0.9255	0.9714	0.6842	0.9286	0.7879	0.9286	0.9250
	ET, GBDT, XGB	0.9145	0.9105	0.5333	0.8889	0.6667	0.8889	0.9176
	ET, RF, XGB	0.9043	0.7562	0.7273	0.5714	0.6400	0.5714	0.9625
	ET, RF, GBDT	0.9362	0.8604	0.7692	0.7692	0.7692	0.7692	0.9630
	ET, RF, GBDT, XGB	**0.9787**	**0.9869**	**0.8182**	**1.0000**	**0.9000**	**1.0000**	**0.9765**
Dataset2	RF, GBDT, XGB	0.9032	0.8932	0.8947	0.9444	0.9189	0.9444	0.8462
	ET, GBDT, XGB	0.8387	0.8979	0.7895	0.9375	0.8571	0.9375	0.7333
	ET, RF, XGB	0.8387	0.8761	0.8824	0.8333	0.8571	0.8333	0.8462
	ET, RF, GBDT	0.9032	0.8310	0.9091	0.9524	0.9302	0.9524	0.8000
	ET, RF, GBDT, XGB	**0.9677**	**0.9949**	**1.0000**	**0.9545**	**0.9767**	**0.9545**	**1.0000**

### Comparison of KISM with Existing state-of-the-art methods

To demonstrate the benefits of KISM’s superiority and robustness, we compared it with other methods on two benchmark datasets.

Since the deep learning model proposed by Talha Burak Alakus et al. [34] only used 18 laboratory features without including age, for the sake of experimental rigor, we also conducted experiments on the KISM model using only the same 18 laboratory features. The results are shown in Table 5, where the bold font represents the best performance. From the table, it is evident that on Dataset1, KISM demonstrates significant advantages over the deep learning and machine learning models proposed by Samin Babaei Rikan et al. [40] and Ahmad A et al. [41]. While KISM has a lower recall rate compared to the work of Talha Burak Alakus et al. [34] and a lower F1-score compared to Su X et al. [42], all other core performance metrics are significantly higher than the other methods. In addition, we validated the improvements in accuracy and AUC of KISM on Dataset2. On Dataset2, each metric of KISM is significantly higher than the Multi-Criteria Decision Making (MCDM) algorithm proposed by Luo J et al [35]. In conclusion, our proposed method has superior performance and powerful generalization capabilities.

**Table 5:** Comparison of the performance of the KISM model on datasets with the state-of-the-art methods.

Dataset	Model	AUC	Accuracy	Sensitivity	Specificity	Precision	Recall	F1-score
Dataset1 without age	KISM	**0.9228**	**0.9894**	**0.8571**	**0.9999**	**0.9999**	0.8571	0.9231
Dataset1	KISM	**0.9869**	**0.9787**	**1.0000**	**0.9765**	**0.8182**	**1.0000**	**0.9000**
Dataset1 without age	Talha Burak Alakus et al. [34]	0.6250	0.8666	----	----	0.8675	**0.9942**	0.9189
Dataset1 without age	Samin Babaei Rikan et al. [40]	0.9220	0.9316	----	0.8500	----	----	----
Dataset1 without age	Ahmad A et al. [41]	0.8890	----	----	----	----	----	----
Dataset1	Ahmad A et al. [41]	0.8920	----	----	----	----	----	----
Dataset1 without age	Su X et al. [42]	0.9100	0.9282	----	----	0.9341	0.9341	**0.9341**
Dataset2	KISM	**0.9949**	**0.9677**	0.9545	1.0000	**1.0000**	0.9545	0.9767
	Luo J et al. [35]	0.8200	0.9300	----	----	----	----	----

### Feature analysis

**Fig 5:**
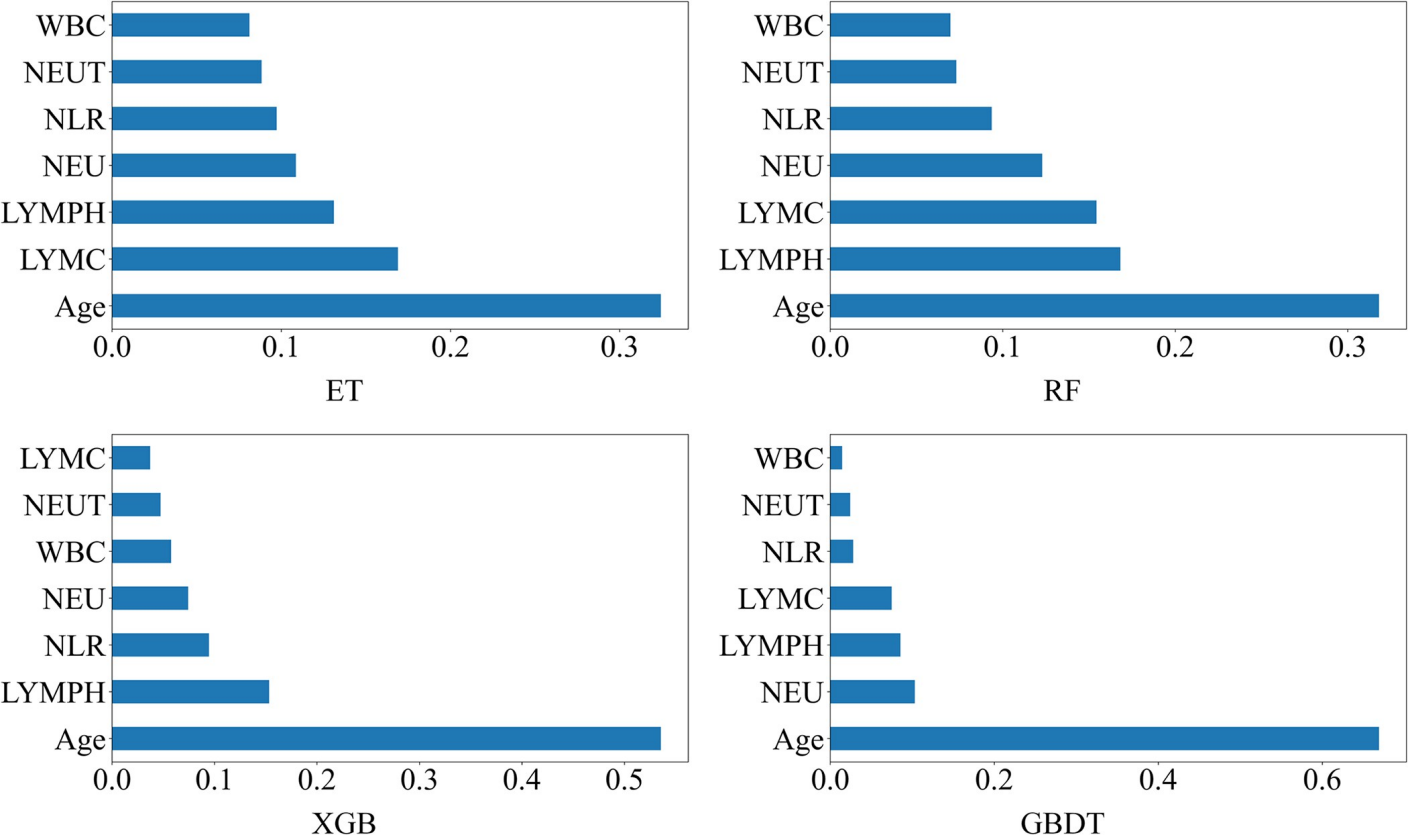
Feature Importance (Feature_Importance_/Dataset2).

**Fig 6:**
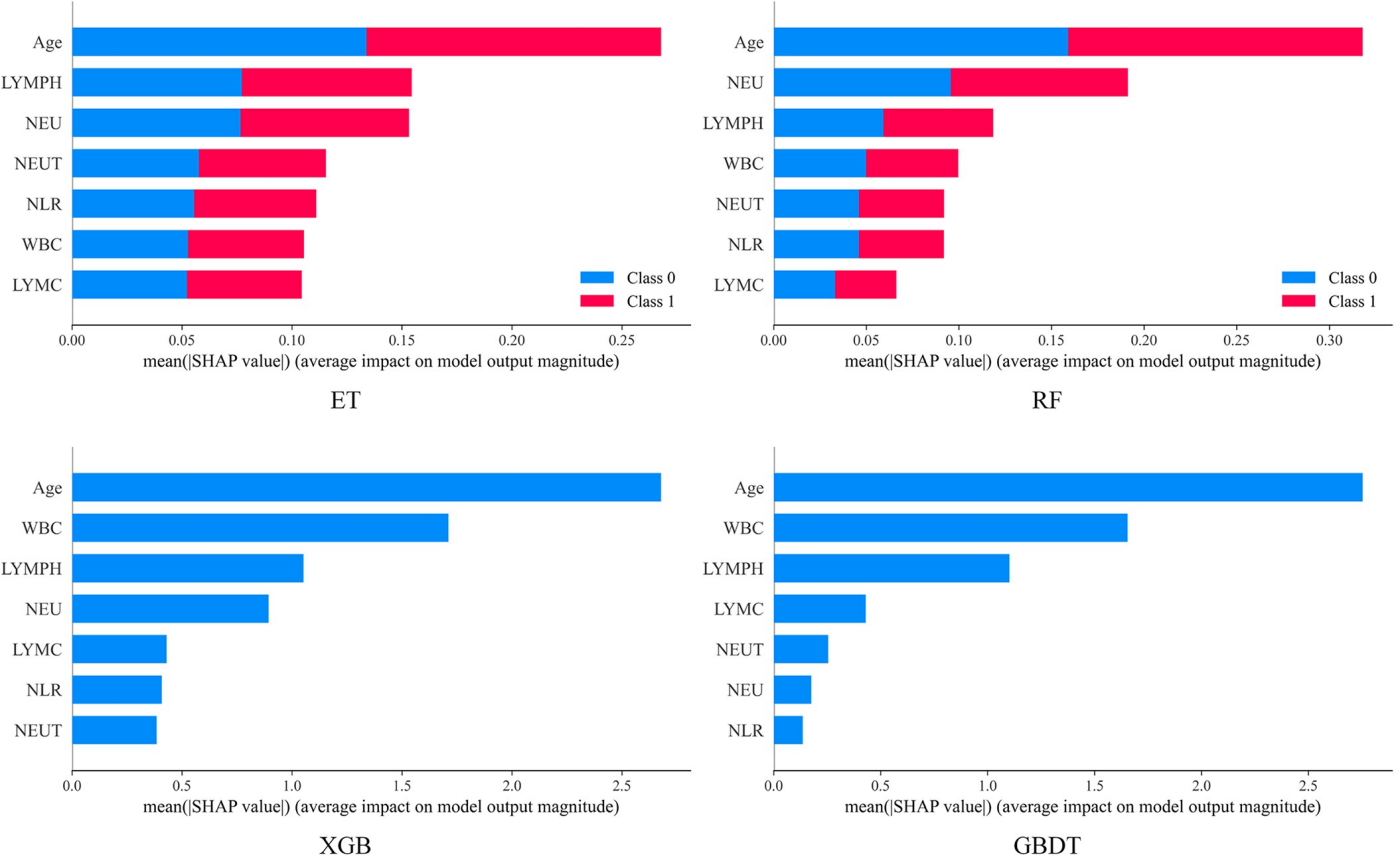
Feature Importance (SHAP/Dataset2).

The ensemble learning model achieves the best performance in KISM, we study the importance of different features in the ensemble model to model performance. Fig 5 shows the importance of features returned by the Feature_Importances_ attribute on Dataset2, with higher Feature_Importances_ representing more important features. Fig 6 is a summation of the SHAP values of all the features of all the samples in Dataset2, which can reflect the feature importance and the contribution of each feature to the positive and negative prediction of the sample, with blue low and red high. The figures related to the importance of all features on Dataset1 are detailed in the S1 and S2 Figs. We can see that on the Dataset1, the top six features in Feature_Importance_ are Leukocytes, Platelets, Proteina C reativa mg/dL, Monocytes, Creatinine, and Eosinophils in that order. At the same time, the most important six in SHAP are Leukocytes, Platelets, Monocytes, Proteina C reativa mg/dL, Eosinophils, and Creatinine. In Feature_Importance_, the top five features are Age, LYMPH, LYMC, NEU, and NLR in order. For SHAP, the top five items are Age, WBC, LYMPH, NEU, and NEUT in order. In conclusion, by interpreting the model trained on Dataset1, we found that Leukocytes, platelets, and Proteina C reativa mg/dL were the most indicative biomarkers for the diagnosis of neointima. Applying the same approach on Dataset2, we further revealed that three important features, Age, LYMPH, and WBC were the most indicative biomarkers for identifying the severity of the patient.

## Conclusion

Early diagnosis and severity prediction of COVID-19 is vital to prevent the spread of COVID-19. Recent studies have shown that blood tests can be used for the initial diagnosis and prognosis of patients with COVID-19 because they are less expensive, faster, and more readily available than other tests. In this study, we proposed a coronavirus classification framework KISM that contributes to the diagnosis and prognosis of COVID-19. The model uses seven machine learning classifier (SVM, ET, RF, GBDT, XGB, LR, and REGX) to improve the overall classifier performance. This model has the following advantages. First, the overall computational cost is low, and the model uses only routine blood test dataset as input for prediction with an integrated model of low complexity. Second, the model can be used as a reliable computational method to assist in the diagnosis and prognosis of COVID-19. In addition, this model is able to output model feature importance scores using Scikit-learn and SHAP, which helps physicians identify biomarkers that are easily overlooked.

Although the KISM framework has shown promising results in COVID-19 diagnosis and prognosis classification, it still has some limitations. Firstly, the two datasets used in this study are relatively small in size, which may limit the performance of the model to some extent. Therefore, future research should focus on using larger and more comprehensive datasets with a greater number of patients to train and test the model. The second limitation is that the scarcity of available datasets. Despite training and using two different datasets in the current research, it is still necessary to explore the generalizability of the model by utilizing more diverse datasets.

## Supporting information

S1 Fig Feature Importance (Feature_Importance_/ Dataset1). (TIF)

S2 Fig Feature Importance (SHAP/ Dataset1). (TIF)
