# Supplementary figures and images for: An interpretable machine learning framework for diagnosis and prognosis of COVID-19
Source: PLoS One. 2023 Sep 21;18(9):e0291961. doi: 10.1371/journal.pone.0291961 (PMC10513274; doi:10.1371/journal.pone.0291961)

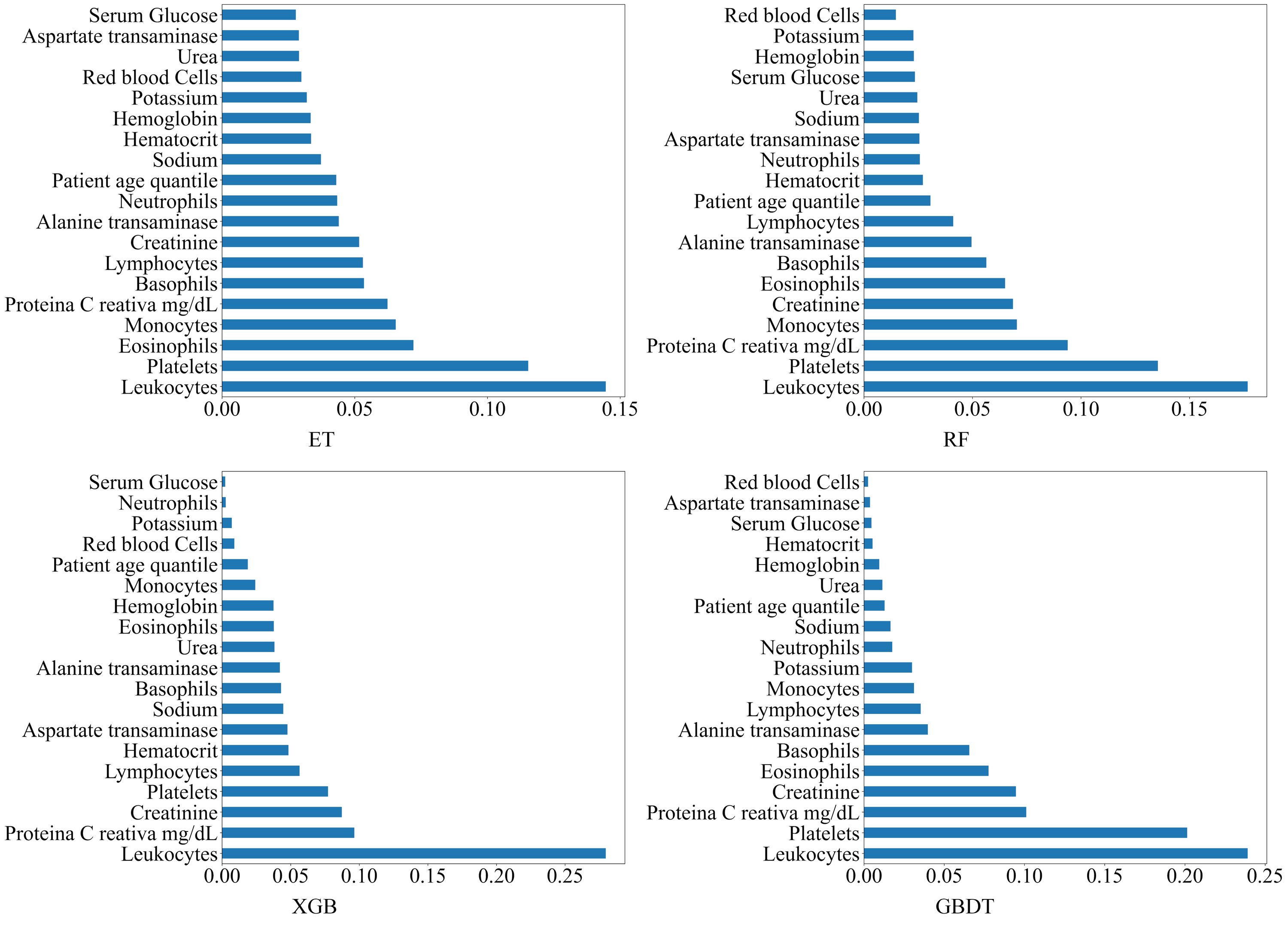

Supplement: S1 Fig — (TIF) [file pone.0291961.s001.tif]

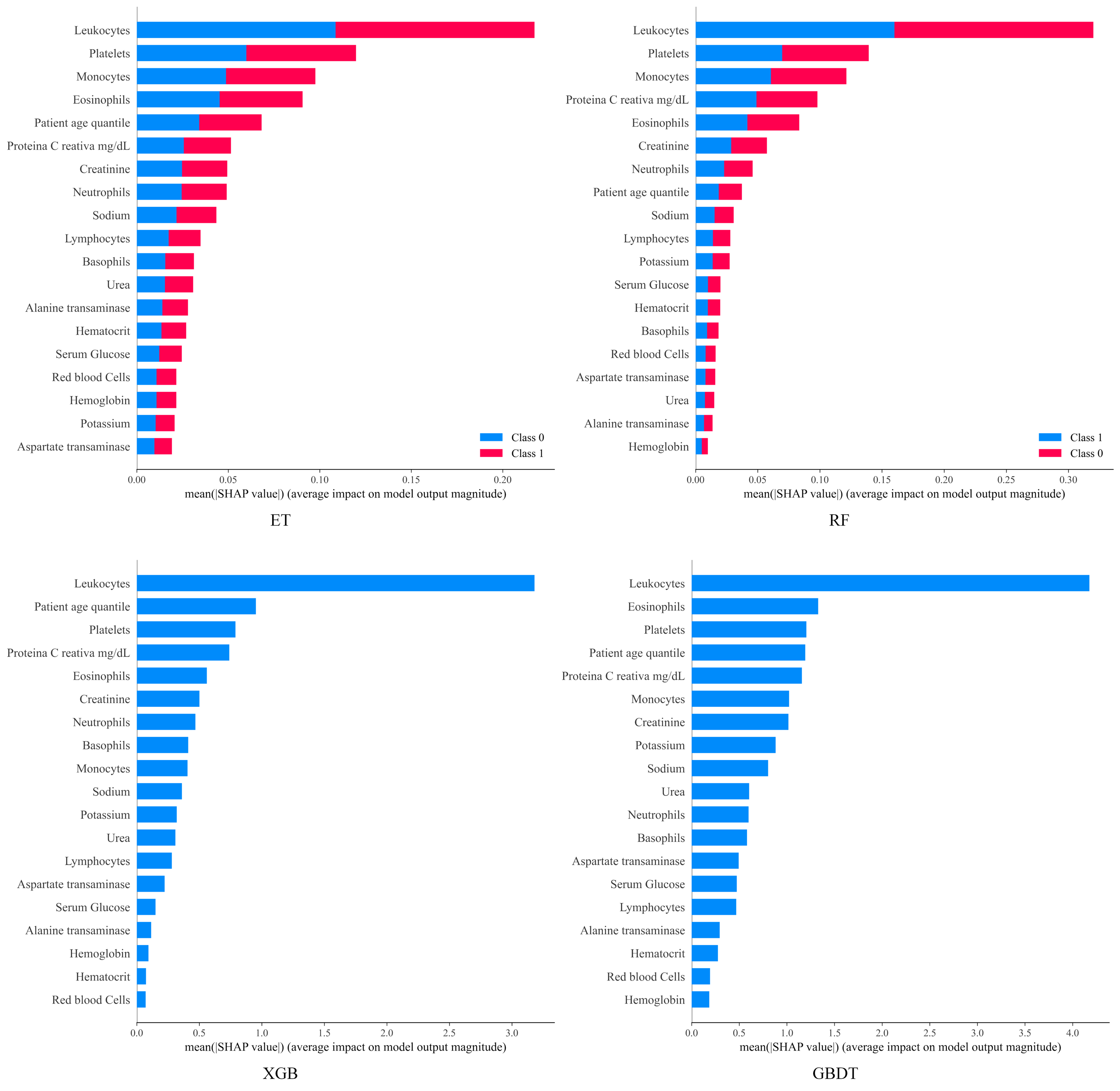

Supplement: S2 Fig — (TIF) [file pone.0291961.s002.tif]
